# Does Early Opioid Use for Relief of Acute Abdominal Pain Increase Use of Abdominal CT or Prolong Emergency Department Length of Stay?

**DOI:** 10.1155/emmi/3925923

**Published:** 2026-07-08

**Authors:** Ben Bloom, Christie Fritz, Shivani Gupta, Jason Pott, Imogen Skene, Raine Astin-Chamberlain, Carlo L. Rosen, Stephen H. Thomas

**Affiliations:** ^1^ Blizard Institute for Neuroscience, Surgery, & Trauma, Barts & The London School of Medicine, London, UK; ^2^ Department of Emergency Medicine, The Royal London Hospital and Barts Health NHS Trust, London, UK; ^3^ Department of Emergency Medicine, Beth Israel Deaconess Medical Center & Harvard Medical School, Boston, Massachusetts, USA

**Keywords:** acute pain, analgesia, emergency department, length of stay, opioid, pain management

## Abstract

**Introduction:**

Opioid use is common for emergency department (ED) patients presenting with undifferentiated abdominal pain (AbdPain). Whether the potential analgesic benefits of opioids are offset by prolonged ED length of stay (LOS)—possibly due to extended evaluations or increased diagnostic testing—remains unclear. The primary objective was to determine whether administration of an opioid, compared to a nonopioid drug, as initial analgesia in AbdPain patients is associated with longer ED LOS after adjusting for demographics and pain severity. Secondary objectives evaluated whether initial opioid use was associated with higher adjusted likelihood of ordering abdominal CT (CTabd) or white blood cell count (WBC) testing.

**Methods:**

This retrospective observational study analyzed 4 months of electronic health record data from a single UK health system, including consecutive adult ED patients presenting with AbdPain who received analgesia and had a documented pain score. Variables included demographic factors, initial pain level, and analgesic administered. ED workup variables included diagnostic testing (CTabd, WBC), LOS, and disposition. Categorical data were summarized as proportions with binomial exact 95% confidence intervals (CIs); continuous data were summarized with medians and interquartile ranges (IQRs). Univariable analyses used Wilcoxon rank‐sum or Pearson’s *χ* ^2^ tests. Multivariable analyses employed quantile median regression for LOS and logistic regression for secondary outcomes.

**Results:**

During the study period, there were 884 patients meeting eligibility criteria. The median ED LOS was 7.7 h (IQR 5.0 to 11.9). CTabd and WBC were ordered in 362 (41.0%) and 797 (90.2%) of cases. Univariable analysis showed no association between initial opioid use and ED LOS (*p* = 0.255), CTabd ordering (*p* = 0.404), or WBC testing (*p* = 0.820). Multivariable modeling adjusting for operational factors, demographics, and pain score confirmed no significant association between initial opioid use and ED LOS (coefficient 0.18, 95% CI: −0.71–1.08, *p* = 0.684), CTabd (OR 1.12, 95% CI: 0.84–1.50, *p* = 0.430), or WBC testing (OR 0.91, 95% CI: 0.58–1.43, *p* = 0.698).

**Conclusions:**

Among ED patients with abdominal pain, initial opioid administration was not associated with longer LOS or increased use of diagnostic testing.

## 1. Introduction

Pain relief is a priority in the emergency department (ED), and patients with chief complaint of abdominal pain (AbdPain) represent a large number of ED cases. ED treatment of AbdPain has historically been marked by issues ranging from opioids’ risks of “masking of exam findings” [1, 2] to cultural biases influencing analgesia timing or choices [3–5].

One facet of AbdPain treatment that has received relatively less attention is the question as to whether analgesia agent selection influences ED length of stay (LOS) or ED workup. In an era in which healthcare resources are subject to increasing strain, reduced ED LOS is an independently important endpoint [6–9]. In addition to operational ramifications, prolonged ED LOS has been associated with medical consequences ranging from higher geriatric delirium risk [10] to low care quality for AbdPain [11].

For some groups (e.g., back pain and geriatrics), the use of opioid ED analgesia is associated with prolongation of LOS, potentially via a mechanism of increased diagnostic work‐up [9, 12]. The question regarding ED use of initial opioid analgesia is whether emergency physicians are executing a de facto trade‐off, whereby the “cost” of safely effective opioid AbdPain analgesia is increased workup and prolonged ED LOS.

In a set of ED patients with AbdPain chief complaint, we assessed whether—adjusting for factors such as pain severity and demographics—use of opioid (as compared to nonopioid drugs) initial analgesia was associated with the primary endpoint of prolonged ED LOS. Secondary endpoint analyses comprised assessment for association between initial opioid use and likelihood of ordering abdominal computed tomography (CTabd) or white blood cell count (WBC) lab testing.

## 2. Methods

### 2.1. Design and Setting

Prospectively recorded electronic health record (EHR) data from a single United Kingdom (UK) ED was the sole source of information for this retrospective observational cohort study. The National Health Service (NHS) acute trust ethics review process designated the study as a service evaluation.

Data in the EHR reflected four months (December 2022 through March 2023) of consecutive adult (> 17) ED visits, by patients with chief complaint of abdominal pain denoted by the Systemized Nomenclature of Medicine–Clinical Terms (SNOMED‐CT) system’s chief complaint code 21522001. The EDs were variably staffed, with varying physician numbers and shift lengths. For analysis purposes, in order to account for potential circadian variation in ED operations, there were three shifts defined a priori: day (0700–1500), evening (1500–2300), and night (2300‐0700). All care was supervised by board‐certified Emergency Medicine physicians.

### 2.2. Data

This is a subset analysis of a larger study (*n* = 4231) that reported on demographic predictors of time to analgesia [13]. The database was restricted a priori to cases spending less than 24 h in the ED. As reported previously, data were entered into the EHR at the time of patient registration and care and then extracted into a spreadsheet (Excel, microsoft.com) before anonymization for statistical analysis in Stata (Version 18MP, stata.com). The process of extracting and anonymizing identifiable data was conducted by members of the direct care team.

Data definitions were those in use in the study system’s EHR and in the UK NHS data dictionary. Age is reported in bands (18‐29, 30‐39, 40‐49, etc.), sex is recorded as binary, and ethnicity employs both detailed (14‐category) and “high‐level” four‐category schemes. The pain score was recorded using the Numerical Rating Scale which is manually collected by ED staff on an 11‐point scale (0–10).

### 2.3. Endpoints and Statistical Methods

The primary endpoint was ED LOS in the metric of hours. LOS was defined as the duration of time between when patient is registered on the electronic medical record and when patient is discharged (out of hospital or to inpatient space) from the ED recorded in electronic medical record. After quantile‐normal plotting and Shapiro–Wilk testing demonstrated non‐normal distribution, central tendency was described with medians with bootstrapped 95% confidence intervals (CIs), with dispersion described using interquartile range (IQR).

Categorical variables were described using proportions (with binomial exact 95% CIs) and assessed for association using *χ* ^2^ or Fisher’s exact test (if cell values fell below five). Nonparametric Wilcoxon rank‐sum (for two groups) or Kruskal–Wallis (for multiple groups) testing was used for assessing associations between ED LOS and continuous or ordinal predictors. For ordered‐group variables (age group), univariate analysis included nonparametric trend evaluations employing the Cochran–Armitage approach to identify linear and nonlinear trends.

Multivariable modeling for the primary (ED LOS) and secondary (CTabd, WBC) variables evaluated operational (e.g., study month, shift time, and day of week) and demographic (age, sex, and ethnicity) factors, as well as the clinical variable of initial pain score. Modeling proceeded using a stepwise approach, with univariable *p* cutoff of 0.20 used to determine eligibility for assessment in multivariable modeling [14].

Given its expected (and confirmed) marked non‐normality, the continuous endpoint ED LOS was evaluated using quantile (median) regression; this approach assesses median ED LOS, conditional on values of independent variables. The Machado–Santos Silva test for heteroskedasticity was used to confirm preferability of quantile over linear ordinary least‐squares regression, and the Parente–Santos Silva test for intracluster correlation was used to evaluate whether the quantile regression required clustering [15]. Since traditional linear regression metrics such as *r* ^2^ are less useful (or not calculated) in quantile regression, postestimation model evaluation was performed by assessing specificity (link test) [15].

The secondary‐variable dichotomous endpoints (CTabd and WBC ordering) were evaluated with multivariable logistic regression and odds ratios (ORs). Covariate inclusion and significance were based on the likelihood ratio test, and model performance was evaluated using Akaike and Bayesian information criteria. Postestimation model evaluation included assessment of specification (link test hat^2^), discrimination (c statistic), and calibration (Hosmer–Lemeshow goodness‐of‐fit test) [16, 17].

## 3. Results

During the four‐month study period, there were 884 patients meeting eligibility criteria. Table 1 presents characteristics of the patients. Additional patient details (e.g., full 14‐category ethnicity and listing of ED discharge diagnoses) are found in the Supporting Information (available here).

**TABLE 1:** Study population characteristics.

Parameters	*n* = 884
Age	
18–29	219 (24.8%)
30–39	182 (20.6%)
40–49	154 (17.4%)
50–59	125 (14.1%)
60–69	92 (10.4%)
70–79	59 (6.7%)
80–89	43 (4.9%)
90+	10 (1.1%)
Female sex	511 (57.8%)
High‐level (4‐category) ethnicity	
Asian	297 (33.6%)
Black	100 (11.3%)
White	368 (41.6%)
Other	119 (13.5%)
Initial pain score (0–10 scale)	
Mild (0–3)	355 (40.2%)
Moderate (4–7)	325 (36.8%)
Severe (8–10)	204 (23.1%)
Emergency department workup	
Hematology testing (WBC)	797 (90.2%)
Chemistry laboratory testing	763 (86.3%)
Urine laboratory testing	790 (89.4%)
Abdominal CT scan	362 (41.0%)
Any consultation (including surgical)	577 (65.3%)
Surgical service consultation	392 (67.9%)
Disposition	
Admission (to any service)	477 (54.0%)
Admission to surgical service	157 (17.8%)
ED diagnoses documented in > 2% of patients	
Abdominal pain	252 (28.5%)
No abnormality detected	44 (5.0%)
Vomiting and/or diarrhea	42 (4.8%)
Biliary tract pain	39 (4.4%)
Renal colic	38 (4.3%)
Venous thromboembolism risk	35 (4.0%)
Pancreatitis	32 (3.6%)
Gastritis and/or reflux	30 (3.4%)
Appendicitis	29 (3.3%)
Urinary tract infection	29 (3.3%)
Bowel obstruction	26 (2.9%)
Endometriosis/endometritis	20 (2.3%)
Constipation	19 (2.2%)

*Note:* Data are presented as *n* unless indicated otherwise.

Abbreviations: CT, computed tomography; ED, emergency department; WBC, white blood cell count.

For the overall set of 884 patients, the median ED LOS was 7.7 h (IQR 5.0–11.9). CTabd and WBC were ordered in 362 (41.0%) and 797 (90.2%) of cases, respectively. Table 2 depicts univariable analyses—all nonsignificant—for association of initial opioid analgesia with ED LOS as well as the secondary endpoints.

**TABLE 2:** Univariable analysis: Initial abdominal pain analgesia, length of stay, and ordering of abdominal CT imaging or white blood cell count.

Initial analgesia	Median hours ED LOS	Abdominal CT ordered	WBC ordered
Includes opioid (*n* = 376, 42.5% of 884)	7.6 (95% CI 6.8–8.4, IQR 4.5–11.8)	160 of 376 (42.6%, 37.5%–47.7%)	338 of 376 (89.9%, 86.4%–92.7%)
Does not include opioid (*n* = 508)	7.8 (95% CI 7.2–8.2, IQR 5.2–12.0)	202 of 508 (39.8%, 35.5%–44.2%)	459 of 508 (90.4%, 87.4%–92.8%)
*p*, opioid *vs.* nonopioid	0.255	0.404	0.820
Effect estimate, opioid‐associated increase	−0.3 (95% CI ‐0.2–0.9)	Risk ratio 1.07 (0.91–1.25)	Risk ratio 0.99 (0.95–1.04)

*Note:* IQR, interquartile range.

Abbreviations: CI, confidence interval; CT, computed tomography; ED, emergency department; LOS, length of stay; WBC, white blood cell count.

In order to account for factors (besides analgesia care) that could affect ED LOS and diagnostic and laboratory test ordering, multivariable quantile regression was executed. All variables were assessed for univariable association with the primary and secondary endpoints, with detailed results presented in the Supporting Information.

For the primary endpoint of ED LOS, variables incorporating in model evaluation included study month, weekday, shift time, age group, initial pain score, and ethnicity. Modeling produced a final model which incorporated the dichotomous predictor of initial analgesia with an opioid, as well as the following LOS‐associated covariates: month, shift, age group, initial pain score, and ethnicity. In this final model, the details of which are presented in the Supporting Information, all covariates except for initial opioid analgesia were statistically significant. The nonsignificant findings regarding ED LOS prolongation by use of an opioid initial analgesic are summarized in Table 3. Full details on the final model and its postestimation evaluation are provided in the Supporting Information.

**TABLE 3:** Multivariable results: initial opioid analgesia and ED LOS and for ordering of abdominal CT or white blood cell count.

Endpoint	Initial opioid analgesia: Multivariable point estimate (95% CI, *p*)
Emergency department length of stay	*β* = 0.18 h (−0.71 to 1.08, *p* = 0.684)
Ordering of abdominal CT scan	Odds ratio 1.12 (0.84–1.50, *p* = 0.430)
Ordering of white blood cell count	Odds ratio 0.91 (0.58–1.43, *p* = 0.698)

For the secondary endpoint of CTabd ordering, variables incorporating in model evaluation included study month, sex, age group, initial pain score, and ethnicity. Modeling produced a final model which incorporated the dichotomous predictor of initial analgesia with an opioid, as well as the following CTabd‐associated covariates: sex, age group, and initial pain score. In this final model, the details of which are presented in the Supporting Information, all covariates except for initial opioid analgesia were statistically significant. Table 3 outlines nonsignificant findings regarding increased odds of CTabd ordering associated with use of an opioid initial analgesic. Full details on the final model and its postestimation evaluation are provided in the Supporting Information.

For the secondary endpoint of WBC ordering, variables incorporating in model evaluation included study month, age group, and initial pain score. Modeling produced a final model which incorporated the dichotomous predictor of initial analgesia with an opioid, as well as the following WBC‐associated covariates: age group and initial pain score. In this final model, the details of which are presented in the Supporting Information, both covariates (but not initial opioid analgesia) were statistically significant. Table 3 outlines nonsignificant findings regarding increased odds of WBC ordering associated with use of an opioid initial analgesic. Full details on the final model and its postestimation evaluation are provided in the Supporting Information.

## 4. Discussion

This study examined whether giving opioids as first‐line analgesia to ED patients with abdominal pain was associated with additional diagnostic workup or a longer ED LOS. In both univariable models and analyses adjusted for relevant covariates, early opioid dosing showed no association with increased use of computed tomography (CT), ordering of WBC, or extended LOS. Many emergency clinicians continue to worry that opioids given for undifferentiated abdominal pain could obscure examination findings, prompt additional testing, or slow diagnosis and disposition. However, in this cohort, initiating opioid analgesia early in the ED course did not appear to meaningfully affect downstream diagnostic decisions or operational endpoints. The diagnostic approach to abdominal pain in the ED is typically driven by the presenting complaint and associated symptoms, physical examination findings, patient age and comorbidity burden, and the clinician’s concern for surgical pathology, factors that often outweigh the specific analgesic given or the dose selected. For many patients with abdominal pain, CT imaging and laboratory studies are ordered early in the evaluation, in some cases before analgesia is administered, which would limit the extent to which opioids alone could shift diagnostic decision‐making. Also, growing emphasis on pain control and timely analgesia likely has increased clinician comfort with opioids when used in appropriately selected ED patients.

Our findings are consistent with expert reviews on ED pain management emphasizing abdominal pain as an indication for opioid administration [18]. This may be hindered by a study with data from the US National Hospital Ambulatory Medical Care Survey (NHAMCS) that demonstrated that, for ED patients with low back pain, there is association between use of opioids (*vs.* nonopioid analgesia) and prolonged ED LOS [9]. This was further borne out in a recent Australian study, also assessing low back pain but focusing on geriatric patients, which found that opioids were the most commonly used treatment (administered in over two‐thirds of patients) and that opioid treatment was associated with a significant increase in ED LOS [12]. This could be attributable to differences in the system or to differences in diagnostic approach, workup, and disposition for back pain compared with abdominal pain. In Germany, care‐quality assessments have concluded that abdominal pain cases with extended ED LOS (defined as over six hours) had worse outcomes, with the authors recommend a target ED LOS of < 360 min [11]. Other investigators focusing on geriatric patients have not identified either ED opioid administration or increased ED LOS as being independently associated with increased delirium risk [10] which may help to reduce provider qualms about aggressive analgesia strategy. In an age where LOS is increasingly scrutinized and is known to increase patient morbidity and mortality, any factor that would increase LOS, such as potentially opioid pain control, would be seen as extremely undesirable. Encouragingly, a large‐scale study from Canada reported that earlier time to initial analgesia, but not pain relief itself, was associated with shorter ED LOS(8). Differences in healthcare systems, patient populations, and prescribing practices may contribute to the disparities in these studies, but our study contributes to the evidence that appropriate pain control does not increase ED LOS or additional diagnostic testing.

In a large North American study [18], it was previously found that 74% of patients were discharged from the emergency setting on moderate to severe pain and that many patients do not feel comfortable voicing their desire for analgesia. This underuse of pain control in the ER is especially concerning, combined with the knowledge that inadequate pain control increases morbidity and mortality measures.This is prominent especially related to quality of life, poor physician functioning, and the knowledge that inadequate treatment of acute pain is significantly associated with the development of chronic pain [19].

Understanding of problems associated with ED analgesics’ side effects (particularly those of opioids) has spurred assessment of emphasizing nonopioid analgesia in ED cases (e.g., trauma [18]) and even nondrug pain management modalities (e.g., transcutaneous electrical nerve stimulation for abdominal pain) [19]. Despite this, and also associated with the significant risks of inadequate acute pain control, opioids remain the treatment of choice, and recommendation for the treatment of moderate to severe pain in the emergency setting. There are numerous barriers to clinicians’ ordering of opioid pain medications, which include a lack of training around and comfort with opioid side effects, concern for side effects, legal or regulatory concerns as well as concerns around addiction [19].

Viewed through the lens of ED operations, our results offer some reassurance to clinicians and leadership as they try to reconcile adequate analgesia with the increasing pressure to maintain throughput targets. While the design does not permit causal inference, the data indicate that selecting an opioid as the first‐line analgesic is not, on its own, associated with prolonged LOS or increased diagnostic testing in this cohort.

### 4.1. Limitations

This study was conducted in a single hospital system in a single geographic area, which may limit the generalizability of the findings. In addition, most patients underwent laboratory testing (e.g., over 90% had a WBC). This high baseline rate of laboratory utilization may have limited our ability to detect an association between opioid administration and WBC ordering. However, the near‐universal use of laboratory testing also suggests that testing decisions were likely independent of the choice of initial analgesia.

We assessed the association of initial analgesia with test ordering. We did not assess or draw conclusions regarding whether testing was under‐ or overutilized. Our data merely demonstrate that the testing decisions that were made were made independent of which initial analgesia was administered.

The study database was restricted a priori to including only those patients with ED stays of up to 24 h. Any results drawn from our analysis should not be extrapolated to patients in the ED for more than a full day.

We included in the study only those patients who received an analgesic and also had at least an initial pain score recorded. This decision was made during study planning to ensure the ability to adjust for pain severity’s important contribution to ED pain care.

Finally, this study evaluated only the impact of opioids administered as initial analgesic administered. We did not assess whether opioid administration later during the ED stay influenced LOS or the likelihood of abdominal CT imaging.

## 5. Conclusion

In this large case series, selection of opioids for initial pain control was not associated with increased ED LOS, which, when prolonged, has been shown to lead to worse patient‐centered outcomes. Encouragingly, providing opioid analgesia as the first pain medication administered also did not increase the provider’s likelihood of ordering CT imaging with its attendant risks. These findings suggest that concerns regarding prolonged ED LOS or increased radiologic testing should not preclude appropriate use of opioid analgesia for initial pain management in ED patients presenting with acute abdominal pain.

## Author Contributions

The study was conceived by Ben Bloom. Data acquisition was led by Ben Bloom with collection, organization, and tabulation of data executed by Shivani Gupta, Jason Pott, Imogen Skene, and Raine Astin‐Chamberlain. Data analysis was led by Stephen H. Thomas with assistance from Carlo L. Rosen. The manuscript was drafted by Carlo L. Rosen, Stephen H. Thomas, and Christie Fritz and with critical revisions by Ben Bloom.

## Funding

There are no sources of funding to declare.

## Disclosure

All authors have agreed on the final version of this manuscript.

## Consent

The authors have nothing to report.

## Conflicts of Interest

The authors declare no conflicts of interest.

## Supporting Information

Additional supporting information can be found online in the Supporting Information section.

## Supporting information


**Supporting Information** S1: Details of patients and pain medication. S2: Model‐building and postestimation evaluation. S3: Sensitivity analysis—ethnicity categorization.

## Data Availability

The datasets used and/or analyzed during the current study are available from the corresponding author on reasonable request.
