# Supplementary material for: Does Early Opioid Use for Relief of Acute Abdominal Pain Increase Use of Abdominal CT or Prolong Emergency Department Length of Stay?
Source: Emerg Med Int. 2026 Jul 8;2026:3925923. doi: 10.1155/emmi/3925923 (PMC13343369; doi:10.1155/emmi/3925923)
Supplement: Supplementary file 1 — Supporting Information S1: Details of patients and pain medication. S2: Model‐building and postestimation evaluation. S3: Sensitivity analysis—ethnicity categorization. [file EMMI-2026-3925923-s001.docx]

**SUPPLEMENT**

**S1: Details of patients and pain medication**

**S2: Model-building and post-estimation evaluation**

**S3: Sensitivity analysis – ethnicity categorization**

**S1: Details of patients and pain medication**

*Extended results on patient characteristics*

Ethnicity for the complete NHS categorization is depicted in Table S1-1. The table also depicts the ED discharge diagnoses that were seen in at least 1% of cases but less commonly than the 2% threshold for reporting in the main results.

**Table S1-1.** Details on patients (*n* = 884)

| **Parameter** | ***n* (% of 884)** |
| --- | --- |
| Detailed (14-category) ethnicity |  |
| Asian: Bangladesh | 198 (22.4%) |
| Asian: China | 18 (2.0%) |
| Asian: India | 24 (2.7%) |
| Asian: Pakistan | 11 (1.2%) |
| Asian: Other | 46 (5.2%) |
| Black: Africa | 51 (5.8%) |
| Black: Caribbean | 30 (3.4%) |
| Black: Other | 19 (2.2%) |
| White: Britain | 250 (28.3%) |
| White: Ireland | 11 (1.2%) |
| White: Other | 107 (12.1%) |
| Other single ethnicity | 73 (8.3%) |
| Mixed ethnicity | 16 (1.8%) |
| No ethnicity indicated | 30 (3.4%) |
|  |  |
| ED diagnoses documented in 1-2% of patients |  |
| Renal (non-colic) pain | 16 (1.8%) |
| Cardiac/chest pain | 15 (1.7%) |
| Crohn’s Disease or ulcerative colitis | 15 (1.7%) |
| Neurologic pain | 15 (1.7%) |
| Hernia | 13 (1.5%) |
| Obesity | 13 (1.5%) |
| Ovarian pain | 13 (1.5%) |
| Hepatic disease | 11 (1.2%) |
| Back pain | 10 (1.1%) |

*Extended results on administrations of various drugs/combinations*

For hypothesis-generating purposes, since there are relatively few large-scale analyses of single-center ED approaches to treating abdominal pain, Table S1-2 provides detailed information on specific analgesics administered. Medications were considered administered simultaneously if they were given within one minute of each other; in such cases the multi-drug regimen is reflected in listing of more than one drug in a single dosing. One drug, codeine, was often administered in a single-pill combination with paracetamol.

In 884 patients with overall (*i.e.* during entire ED stay) 2721 AbdPain analgesia dosings – monotherapy or simultaneously administered multi-therapy – the most common single-agent treatment was paracetamol, administered in 1419 (52.1%) dosings. The next most common AbdPain analgesic was morphine, used as single-drug therapy 629 times (23.1% of 2721 dosings) and as part of multi-drug therapy an additional 77 times (2.8% of 2721). One other opioid, codeine, was used as monotherapy (281 times, 10.3% of 2721) or as part of multi-drug regimens (123 times, 4.5% of 2721).

The time interval between initial and second doses of analgesia was substantial. The median elapsed time from initial to follow-up analgesia was 145 minutes (95% CI 123-165), with IQR 56 to 320 minutes.

**Table S1-2.** Details on abdominal pain analgesia administered

|  | **Abdominal pain analgesia administration** | | | | |  |  |
| --- | --- | --- | --- | --- | --- | --- | --- |
|  | **1st dosing** | **2nd dosing** | **3rd dosing** | **4th dosing** | **5th dosing** | **Total** | **% of 2721** |
| Non-opioids: Single-agent Rx |  |  |  |  |  |  |  |
| diclofenac | 19 | 19 | 14 | 7 | 2 | 61 | *2.2%* |
| hyoscine | 16 | 21 | 18 | 6 | 7 | 68 | *2.5%* |
| ibuprofen | 15 | 13 | 9 | 8 | 7 | 52 | *1.9%* |
| paracetamol (acetaminophen) | 440 | 260 | 243 | 240 | 236 | 1419 | *52.1%* |
|  |  |  |  |  |  |  |  |
| Non-opioids: Multi-agent Rx | |  |  |  |  |  |  |
| diclofenac/paracetamol | 7 |  |  | 2 |  | 9 | *0.3%* |
| hyoscine/paracetamol | 2 |  | 1 | 1 |  | 4 | *0.1%* |
| ibuprofen/paracetamol | 9 | 1 | 1 |  |  | 11 | *0.4%* |
|  |  |  |  |  |  |  |  |
| Opioids: Single-agent Rx |  |  |  |  |  |  |  |
| codeine | 89 | 98 | 43 | 32 | 19 | 281 | *10.3%* |
| morphine | 202 | 208 | 121 | 61 | 37 | 629 | *23.1%* |
|  |  |  |  |  |  |  |  |
| Opioid-including combinations | |  |  |  |  |  |  |
| codeine/diclofenac | 1 |  |  | 1 |  | 2 | *0.1%* |
| codeine/ibuprofen | 1 | 1 | 1 | 2 |  | 5 | *0.2%* |
| codeine/paracetamol | 49 | 20 | 19 | 9 | 3 | 100 | *3.7%* |
| codeine/hyoscine/paracetamol |  | 1 |  |  |  | 1 | *0.0%* |
| codeine/ibuprofen/paracetamol | 2 |  |  |  |  | 2 | *0.1%* |
| morphine/codeine |  | 5 | 6 | 2 |  | 13 | *0.5%* |
| morphine/diclofenac | 2 |  | 2 | 1 |  | 5 | *0.2%* |
| morphine/hyoscine | 2 | 1 | 1 | 2 |  | 6 | *0.2%* |
| morphine/paracetamol | 28 | 6 | 12 | 7 |  | 53 | *1.9%* |
|  |  |  |  |  |  |  |  |
| *Totals* | *884* | *654* | *491* | *381* | *311* | *2721* | *100.0%* |

**S2: Model-building and post-estimation evaluation**

*Univariable assessment of variables associated with ED LOS or obtaining CT scan*

For assessment of the variables potentially contributing to the primary or secondary endpoints, univariable analyses were executed (Table S2-1). Variables such as test ordering (*e.g.* laboratory, CT) were not included in Table S2-1, since such variables could be considered as influenced by opioid administration (*i.e.* more testing done since opioids were administered). Table S2-1 variables found to have univariate *p* <.20 were included by a priori study planning, in the assessment variable list for multivariable models.

**Table S2-1.** Univariable analyses to identify covariates in models for primary endpoint (ED LOS) and secondary endpoint (ordering of CT scan of abdomen)

| **Variable** | ***p*, association with ED LOS** | **Evaluate in ED LOS model** | ***p*, association with CT** | **Evaluate in CT model** | ***p*, association with WBC** | **Evaluate in WBC model** |
| --- | --- | --- | --- | --- | --- | --- |
| Study day | .236 | No | .281 | No | .278 | No |
| Month | .007 | Yes | .050 | Yes | .097 | Yes |
| Day of week | .010 | Yes | .471 | No | .725 | No |
| Shift | <.001 | Yes | .288 | No | .793 | No |
| Sex | .678 | No | <.001 | Yes | .871 | No |
| Age group | <.001 | Yes | <.001 | Yes | .062 | Yes |
| Initial pain score | .001 | Yes | .061 | Yes | .022 | Yes |
| Ethnicity | .001 | Yes | .004 | Yes | .677 | No |
| Non-English primary language | .932 | No | .526 | No | .728 | No |

*Final detailed model: ED LOS*

The table below demonstrates the full and final model for the primary endpoint. The table includes the primary predictor variable (initial therapy with an opioid analgesic), although this variable was non-significant.

**Table S2-2**. Detailed model: ED LOS

| **Independent variable** | **β (95% CI, *p*)** |
| --- | --- |
| Use of an opioid as initial analgesic | 0.18 (-0.71 to 1.08, *p* = .684) |
| Month (December 2021 baseline) |  |
| January 2022 | 0.06 (-0.97 to 1.08, *p* = .912) |
| February 2022 | *1.48 (0.36-2.60, *p* = .010) |
| March 2022 | *1.36 (0.17-2.56, *p* = .025) |
| Shift (day shift 0700-1500 baseline) |  |
| Evening (1500-2300) | *1.29 (0.45-2.13, *p* = .003) |
| Night (2300-0700) | 0.69 (-0.85 to 2.22, *p* = .380) |
| Age group (each unit increase in decade) | *0.44 (0.19-0.69, *p* = .001) |
| Initial pain score (on 0-10 scale) | *-0.22 (-0.35 to -0.09, *p* = .001) |
| Detailed ethnicity (“not indicated” baseline) |  |
| Asian: Bangladesh | 0.00 (-2.51 to 2.52, *p* = .998) |
| Asian: China | 0.28 (-3.92 to 4.49, *p* = .894) |
| Asian: India | 1.33 (-2.16 to 4.82, *p* = .454) |
| Asian: Pakistan | 1.16 (-1.92 to 4.24, *p* = .460) |
| Asian: Other | 0.03 (-2.69 to 2.74, *p* = .983) |
| Black: Africa | 1.53 (-1.85 to 4.91, *p* = .375) |
| Black: Caribbean | 1.44 (-1.62 to 4.49, *p* = .357) |
| Black: Other | 2.52 (-2.66 to 7.69, *p* = .340) |
| White: Britain | -0.74 (-3.27 to 1.78, *p* = .563) |
| White: Ireland | *3.86 (0.71-7.01, *p* = .016) |
| White: Other | -0.14 (-2.92 to 2.65, *p* = .923) |
| Other single ethnicity | 0.57 (-2.14 to 3.27, *p* = .681) |
| Mixed ethnicity | 3.21 (-1.45 to 7.86, *p* = .177) |

**p* < .05

*Final detailed model: CTabd*

The table below demonstrates the full and final model for the primary endpoint. The table includes the primary predictor variable (initial therapy with an opioid analgesic), although this variable was non-significant.

**Table S2-3**. Detailed model: Ordering of abdominal CT

| **Independent variable** | **β (95% CI, *p*)** |
| --- | --- |
| Use of an opioid as initial analgesic | 1.12 (0.84-1.50, *p* = .430) |
| Sex (female baseline) | *1.79 (1.34-2.39, *p* < .001) |
| Age group (each unit increase in decade, linear term) | *2.13 (1.56-2.92, *p* < .001) |
| Age group (quadratic term) | *0.95 (0.91 to 0.98, *p* = .006) |
| Initial pain score (on 0-10 scale) | *1.08 (1.03-1.13, *p* = .001) |

**p* < .05

*Final detailed model: WBC*

The table below demonstrates the full and final model for the secondary endpoint of ordering WBC. The table includes the primary predictor variable (initial therapy with an opioid analgesic), although this variable was non-significant. Of note, using AIC/BIC criteria the initial pain score was retained in the model despite a non-significant *p* value; inclusion or exclusion of the initial pain score variable did not result in substantial change to the (non-significant) results for the primary independent variable of initial opioid analgesia.

**Table S2-4**. Detailed model: Ordering of WBC testing

| **Independent variable** | **β (95% CI, *p*)** |
| --- | --- |
| Use of an opioid as initial analgesic | 0.91 (0.58 to 1.43, *p* = .698) |
| Age group (each unit increase in decade) | *1.15 (1.01-1.31, *p* = .033) |
| Initial pain score (on 0-10 scale) | 1.07 (0.99 to 1.14, p = .07) |

**p* < .05

*Post-estimation model evaluation: ED LOS quantile regression*

The first step in post-estimation model evaluation was assessment for misspecification, using the link test. The link-test hat^2^ was non-significant (*p* = 1.000).

An additional step was taken to assess whether the use of opioid initial analgesia had significant influence on ED LOS at quantiles other than the median. The ED LOS range was categorized into thirds, and the quantile regression model was run on the corresponding quantiles of the 33^rd^ and 67^th^ percentiles (rather than the median’s 50^th^ percentile). In these analyses, the opioid analgesia predictor remained non-significant at both the 33^rd^ percentile (coefficient -0.25, 95% CI -0.88 to 0.38, *p* = .435) and at the 67^th^ percentile (coefficient 0.28, 95% CI -0.82 to 1.39, *p* = .617). The calculations suggested that opioid initial analgesia’s effects were similarly non-associated with ED LOS, across the range of ED LOS data.

*Post-estimation model evaluation: CT ordering*

The logistic regression for CT ordering was generated using stepwise modeling, which yielded the following variables as significantly associated with ordering CT: male sex, age decade, and initial pain score. In the model, initial opioid treatment was not associated with ordering CT (OR 1.13, 95% CI 0.85-1.51, *p* = .401).

Post-estimation of the above model suggested misspecification (link test hat^2^ *p* = .002). Therefore, age group was assessed as a higher-order (quadratic) term. This correction resulted in acceptable model specification (hat^2^ *p* = .118) and an essentially unchanged non-significant result for initial opioid treatment: OR 1.12, 95% CI 0.84-1.50, *p* = .430).

Further post-estimation evaluation demonstrated acceptable calibration (Hosmer-Lemeshow goodness-of-fit *p* = .687). Discrimination was also judged acceptable (*c* = .70).

*Post-estimation model evaluation: WBC ordering*

The logistic regression for WBC ordering was generated using stepwise modeling, which yielded the following variables as significantly associated with obtaining a WBC: male sex, age decade, and initial pain score. In this model, the initial use of opioid analgesia was not associated with obtaining a WBC (OR 0.92, 95% CI 0.58-1.43, *p* = .698).

Post-estimation evaluation of the above model suggested borderline discrimination (*c* = .58), suggesting it is not likely useful for prediction. The model was found to have acceptable specification (link test hat^2^ *p* = .409) and calibration (Hosmer-Lemeshow goodness-of-fit *p* = .961).

**.**

**S3: Sensitivity analysis – ethnicity definition categories**

Demographics was preferentially assessed using the more granular NHS categorization scheme as previously employed. Collapsing of the ethnicity term to the NHS high-level four-category scheme was executed to determine if this affected results.

Alteration of ethnicity to the high-level variable did not change results of the primary endpoint. The effect of initial opioid analgesia on ED LOS remained non-significant (quantile regression coefficient 0.12, 95% CI -0.77 to 1.01, *p* = .792). Modeling showed acceptable specification (link test hat^2^ *p* = 1.00).

Results of the secondary endpoints were also unaffected when modeling used the high-level ethnicity catgorization. The WBC endpoint modeling eliminated the ethnicity covariate and the sensitivity analysis yielded the same model reported in the main results. For the CT endpoint, the estimate for initial opioid analgesia effect (OR 1.11, 95% CI 0.83-1.49, *p* = 495) remained similar to that reported in the main results. The sensitivity analysis’ post-estimation evaluation suggested borderline specification (hat^2^ *p* = .051) with acceptable discrimination (*c* = .71) and calibration (Hosmer-Lemeshow goodness-of-fit *p* = .756).
